# Previous pulmonary infection impacts thoracoscopic procedure outcomes in patients with congenital lung malformations: a retrospective cohort study

**DOI:** 10.1186/s12931-023-02412-7

**Published:** 2023-04-18

**Authors:** Dengke Luo, Kaisheng Cheng, Miao Yuan, Chang Xu, Taozhen He, Ru Jia, Shiyi Dai, Chenyu Liu

**Affiliations:** Department of Pediatric Surgery, West China Hospital of Sichuan University, No 37, Guo Xue Xiang, Chengdu, 610041 China

**Keywords:** Pulmonary infection, Congenital lung malformations, Conversion, Complication, Thoracoscopic procedures

## Abstract

**Background and objectives:**

Patients with congenital lung malformations (CLMs) are at high risk for developing pulmonary infection. Nonetheless, prophylactic surgical excision of asymptomatic CLMs is controversial and often delayed to symptoms occurring out of concern for potential operative risks. This study aims to evaluate the impact of previous pulmonary infection on the outcome of thoracoscopic procedures in CLMs patients.

**Methods:**

This was a retrospective cohort study of CLMs patients who received an elective operation at a tertiary care center from 2015 to 2019. Patients were divided into pulmonary infection (PI) or non-infection of pulmonary (NPI) groups according to the history of pulmonary infection. Propensity score matching was used to minimize the bias between groups. The primary outcome was conversion to thoracotomy. Postoperative outcomes were compared between patients with and without PI.

**Results:**

We identified 464 patients, of whom 101 had a history of PI. Propensity score matching yielded a well-balanced cohort of 174 patients. PI was associated with higher conversion to thoracotomy (adjusted odds ratio = 8.7, 95% confidence interval, CI, 1.1–71.2, *p* = 0.039), blood loss (*p* = 0.044), and longer operative time (*p* < 0.001), chest tube placement time (*p* < 0.001), length of stay (*p* < 0.001), and postsurgical length of stay (*p* < 0.001).

**Conclusions:**

Elective operation in CLMs patients with a history of PI was associated with an increased risk of conversion to thoracotomy, operative time, blood loss, chest tube placement time, length of stay, and postsurgical length of stay. Elective thoracoscopic procedures in asymptomatic CLMs patients are safe and effective, and earlier surgical intervention may be warranted.

## Background

Congenital lung malformations (CLMs) comprise a variety of lung anomalies, including congenital pulmonary airway malformations, intralobar sequestration, extralobar sequestration, congenital lobar emphysema, and bronchogenic cysts [1]. The prevalence of CLMs is approximately 1 in 2000 to 2500 live births, and CLMs are detected more frequently because of prenatal screening and increased instrument resolution [2]. Although CLMs can produce fetal nonimmune hydrops requiring prenatal intervention, most remain stable during pregnancy and have no symptoms after delivery, which presents the clinicians’ therapeutic dilemma [3].

CLMs are at a high risk of developing pulmonary infection, which has been reported to be as high as one-third [4, 5]. Moreover, patients are at a lifelong risk of infection due to non-functional pulmonary tissue. CLMs patients with pulmonary infection may present with severe dyspnea, hemoptysis, recurrent fever, and cough. Surgical intervention has gained wide acceptance in this patient population [6]. However, there is ongoing controversy about the need for surgical intervention in asymptomatic patients. There are no universally accepted clinical recommendations or practice guidelines formanaging this patient population due to a lack of natural history [7]. Some surgeons choose an expectant management strategy [8, 9], while many others favor prophylactic surgical excision before symptoms appear to prevent late infection [10, 11].

Nevertheless, such decision-making is subjective. One important reason is that the effect of a history of pulmonary infection on the elective operation outcome is scarce, and considerable conclusion variability exists [8, 12–14]. Although it is reasonable to assume that previous pulmonary infection may lead to a higher rate of postsurgical complications, it is unclear to what extent preoperative infection affects the outcome of thoracoscopic surgery. Thus, we conducted a retrospective cohort study to quantify the effect of previous pulmonary infection on the outcome of thoracoscopic procedures.

## Methods

### Study design

This was a single-center retrospective cohort study, and a prospectively collected database of pediatric patients with CLMs seeking surgical intervention at our hospital was reviewed. This study was approved by the institutional review board of our hospital (number 20,191,082), and written informed consent was obtained from legal guardians. Prophylactic surgical excision of asymptomatic CLMs was chosen in our institution. All patients suspected of CLMs were candidates for thoracoscopic procedures. The study’s inclusion criteria were CLMs diagnosed based on the chest enhanced computed tomography (CT) scan and confirmed at post-resection pathology, age from neonate to 14 years old. Exclusion criteria were patients with incomplete data, emergency operations, bronchogenic cysts, or extralobar sequestration that rarely resulted in an infection and were easily resected. Patients were divided into the pulmonary infection (PI) or non-infection of pulmonary (NPI) groups according to the previous pulmonary infection, which was defined as patients who developed respiratory symptoms and were found to have signs of infection at the same location as the malformations on chest radiography or CT by at least two radiologists, and the symptoms of patients in the PI group all alleviated with medication before elective operation. Propensity score matching was used to minimize the bias between groups. Covariates were considered in adjusted analyses, including sex, BMI, age, preterm delivery, year of operation, thoracic surgery history, ASA physical status, disease categories and locations, maximal lesion size on-axis CT scan, incomplete fissure, type of resection, and comorbidities.

### Surgical procedure and postoperative management

All surgeons had more than eight years of experience in thoracoscopic resection, and the thoracoscopic approach was conventionally treated with all CLMs patients. We chose the pulmonary hilum approach, and the detailed operation methods were reported in our previous study [15]. The lesions involved were characterized as single lobe, double lobes, and triple lobes, confirmed by preoperative CT scan and thoracoscopic visualization. Patients were rechecked by low-dose chest CT one day after surgery, and the chest drain tube was displaced on the condition that there was no atelectasis, bleeding, or residual lesions. After leaving the hospital, patients were seen every six months during the first years and every one to two years thereafter, and chest CT was used to detect recurrence or residual lesions.

### Outcomes

The primary outcome was conversion to thoracotomy. Secondary outcomes were operative findings and postoperative outcomes. Operative findings included operative time, estimated blood loss, rate of chest drain tube placement, and transfusion. Postoperative outcomes included chest tube placement time, postsurgical length of stay, hospital stay, and postsurgical complications, which were graded according to the Clavien–Dindo classification [16], including fever, subcutaneous emphysema, bleeding, pneumothorax, prolonged air leakage (> 7 days), atelectasis, and surgical site infection. Outcomes were compared between the IP and NIP groups.

### Subgroup analyses

The patients in the PI group from the matched cohort were classified as short or long according to the course of the disease. Long was defined as more than one year from successful medication to elective operation, while short was defined as less than one year. The primary outcome was compared between subgroups.

### Statistical analysis

We used a propensity score matching (PSM) approach to minimize the bias between groups. The propensity score of each patient was calculated from multivariable logistic regression based on the described covariates. A 1:1 matching technique without replacement was used to match the PI and NPI groups, with a caliper width of 0.02. Standardized differences were used to confirm a good balance of covariates between the matched PI and NPI groups. Patient baseline characteristics of the total cohort and the matched cohort between the NPI and PI groups were compared using standardized differences. A standardized differences of 10% or higher indicates meaningful differences between groups [17]. Categorical data are presented as counts and percentages, while continuous data are presented as the mean average and standard deviation (SD) for normal data or the median and interquartile range (IQR) for skewness distribution data. The Kolmogorov–Smirnov test was used to test distribution. The conversion rate between the two groups in the total cohort of unmatched patients was compared using the χ2 or Fisher’s exact test, as appropriate. After matching, the differences in surgical outcomes between the PI and NPI groups were compared with the McNemar χ2 test for categorical outcomes and paired t test or Wilcoxon signed-rank test for continuous outcomes as appropriate. The Mann–Whitney U test was used to compare chest tube placement time between the PI and NPI groups. Fisher’s exact test was used in subgroup analyses. All analyses were performed using SPSS (version 26.0, IBM, Armonk, New York, USA), and *p* < 0.05 was considered statistically significant.

## Results

A total of 464 patients were enrolled, including 101 patients in the PI group and 363 patients in the NPI group. The median age was eight months (interquartile range, IQR, 6–18 months), and 60.3% were male. After PSM, 87 patients remained in each group. The baseline before and after PSM were compared and were well balanced between the groups (Table 1). Eight and ten patients in the NPI and PI groups, respectively, had comorbidities, including pectus excavatum, congenital diaphragmatic hernia, cryptorchid, atrial septal defect, asthma, urachal cyst, bronchiectasis, congenital midureteral stricture, asthma, pectus carinatum, and hemangioma.

**Table 1:** Patient Characteristics Before and After Propensity Score Matching

	Before matching				After matching				
	NPI (*n* = 363)	PI (*n* = 101)	Standardized differences			NPI (*n* = 87)	PI (*n* = 87)	Standardized differences	
Sex(Female)	139(38.3%)	45(44.6%)	0.128			36(41.4%)	38(43.7%)	0.047	
BMI	16.8 ± 2.2	16.2 ± 2.1	0.312			16.7 ± 2.6	16.5 ± 2.5	0.078	
Age, m, median (IQR)	8(6,12)	26(10,53)	0.913			14(8,30)	17(8,29)	0.070	
preterm delivery	12(3.3%)	3(2.9%)	0.023			3(3.4%)	2(2.3%)	0.066	
Year of operation									
2015	9(2.5%)	15(14.9%)	0.451			8(9.2%)	10(11.5%)	0.076	
2016	38(10.5%)	21(20.8%)	0.286			20(23%)	18(20.7%)	0.056	
2017	89(24.5%)	22(21.8%)	0.064			23(26.4%)	20(23%)	0.079	
2018	148(40.8%)	26(25.7%)	0.325			23(26.4%)	24(27.6%)	0.027	
2019	79(21.8%)	17(16.8%)	0.127			13(14.9%)	15(17.2%)	0.063	
Operation history	1(0.3%)	8(7.9%)	0.391			1(1.1%)	2(2.3%)	0.093	
ASA status									
1	26(7.2%)	2(2.0%)	0.250			3(3.4%)	2(2.3%)	0.066	
2	337(92.8%)	99(98.0%)	0.250			84(96.6%)	85(97.7%)	0.066	
Disease categories									
congenital pulmonary airway malformations	271(74.7%)	89(88.1%)	0.350			74( 85.1%)	75(86.2%)	0.031	
introlobar sequestration	92(25.3%)	11(10.9%)	0.381			13(14.9%)	11(12.6%)	0.067	
congenital lobar emphysema	0	1(1%)	0.142			0(0%)	1(1.1%)	0.149	
Location									
Left	200(55.1%)	37(36.6%)	0.378			39(44.8%)	35(40.2%)	0.093	
Right	163(44.9%)	64(63.4%)	0.378			48(55.2%)	52(59.8%)	0.093	
Lesion involved									
Single lobe	341(93.9%)	90(89.1%)	0.173			80(92.0%)	78(89.7%)	0.080	
Double lobes	21(5.8%)	10(9.9%)	0.153			7(8.0%)	9(10.3%)	0.080	
Triple lobes	1(0.3%)	1(1.0%)	0.087			0	0		
Maximal size, cm, median (IQR)	4(2.2, 5)	5.3(3.6, 6.4)	0.667			4.6(3.4, 5.3)	5(3.6, 6.1)	0.065	
Type of resection									
lobectomy	216(59.5%)	88(87.1%)	0.657			74(85.1%)	76(87.4%)	0.067	
Segmentectomy	137(37.7%)	11(10.9%)	0.658			10(11.5%)	9(10.3%)	0.039	
Lobectomy and Segmentectomy	10(2.8%)	2(2.0%)	0.026			3(3.4%)	2(2.3%)	0.066	
Incomplete fissure	94(25.9%)	35(34.7%)	0.192			27(31.0%)	30(34.5%)	0.075	
comorbidities	21(5.8%)	12(11.9%)	0.216			8(9.2%)	10(11.5%)	0.076	

### Operative findings

For the total cohort of unmatched patients, patients in the PI group had a higher risk of converting to thoracotomy than those in the NPI group (10.9% vs. 0.3%, *p* < 0.001, odds ratio = 63, 95% confidence interval, CI, 8.2–484.5). Details of operative findings and postoperative outcomes in patients after propensity score matching by a history of pulmonary infection are shown in Table 2. In the propensity matched cohort, the conversion rates to thoracotomy were 9.2% and 1.1%, respectively (*p* = 0.039). The adjusted odds ratio was 8.7 (95% CI, 1.1–71.2). One patient in the NPI group converted to thoracotomy for severe pleural adhesion. The reasons for conversion in the PI group were severe pleural adhesion in five cases, complex anatomy in two cases, and hemodynamic instability in one case. There were no deaths in either group. The median operative times in the NPI and PI groups were 49 and 51 min, respectively (*p* < 0.001). The median intraoperative blood loss was 5 and 10 ml, respectively (*p* = 0.044). The median chest tube placement times were 1 and 2 days, respectively (*p* < 0.001). The median length of stay was 6 and 8 days, respectively (*p* < 0.001). The median postsurgical length of stay was 3 and 5 days, respectively (*p* < 0.001). There were no significant differences in the chest drain tube placement rate between the groups.

**Table 2:** Patient Outcomes After Propensity Score Matching by a History of Pulmonary Infection

Outcomes	NPI (*n* = 87)	PI (*n* = 87)	*P* Value	
Conversion to thoracotomy, *n*	1(1.1%)	8(9.2%)		0.039^a^
Operative time, min, median (IQR)	49(40–52)	51(44–67)		< 0.001^b^
Blood loss, ml, median (IQR)	5(3–12)	10(3–20)		0.044^b^
Length of stay, d, median (IQR)	6(5–6)	8(6–11)		< 0.001^b^
Postsurgical length of stay, d, median (IQR)	3(3–3)	5(3–6)		< 0.001^b^
Chest tube placement, *n*	63(72.4%)	66(75.9%)		0.701^a^
Chest tube placement time, d, median (IQR)	1(1–1)	2(1–2)		< 0.001^c^
Clavien-Dindo Complication Grade				
I	30(34.5%)	20(23.0%)		0.002^a^
II	57(65.5%)	66(75.9%)		0.004^a^
III	0	1(1.1%)		
IV	0	0		
Residual lesion, *n*	0	0		

a McNemar χ2 test

b Wilcoxon signed rank test

c Mann-Whitney U test

### Postoperative outcomes

According to the Clavien–Dindo complication grade, 50 patients (28.7%) did not have any postoperative complications. Minor postoperative complications of grades I and II were significantly higher in the IP group (65.5% versus 75.9%, p = 0.004). The complications classified as grade I included subcutaneous emphysema, pneumothorax, and mild fever, which were spontaneously resolved. In contrast, grade II includes fever requiring antibiotic treatment and prolonged air leakage requiring hypertonic glucose pleurodesis via a chest drain tube in one case. Perioperative transfusions were not needed in all patients. Only one patient in the PI group developed major postoperative complications of Clavien–Dindo grade IIIb of atelectasis, which recovered after fiberoptic bronchoscope-assisted sputum suctioning. No patients developed surgical site infection or postsurgical bleeding. There was no lesion recurrence or delayed pneumothorax on a median follow-up of 4 (IQR, 4–5) years.

### Subgroup analyses

There were 19 and 68 patients in the short and long groups, respectively. There was no association between the time of either short or long from a successful medication to operation and the conversion rate to thoracotomy (15.8% versus 7.4%, *p* = 0.364).

## Discussion

This retrospective cohort study characterized the complication rates and short-term outcomes in pediatric patients with CLMs undergoing thoracoscopic procedures. Patients with a history of PI had a higher risk of conversion to thoracotomy even though the symptoms were alleviated after pharmacotherapy. When patients with PI were matched to patients without PI according to their propensity scores, the rate of conversion to thoracotomy and surgical outcomes of operative time, blood loss, chest tube placement time, length of stay, postsurgical length of stay, and postoperative complications continued to be more likely to occur in patients with preoperative PI.

Concurrently, thoracoscopic procedures have become an increasingly popular operative approach for CLMs patients due to improved cosmesis [18]. A recent meta-analysis found fewer post-operative complications, shorter length of hospital stay, but increased operative time in thoracoscopic procedures [19]. However, conflicting clinical results can present in CLMs resections with different operative approaches because of retrospective review limits. A multicenter cohort study showed prolonged mean operative times of 26 min compared with thoracotomy, although intraoperative blood loss, postoperative complications, chest tube duration, or length of stay is not statistically significant [18]. In contrast, a single-center study has shown decreased mean operative time of 13.1 min with thoracoscopic resection, although the difference was not statistically significant. However, the chest tube stay and length of stay were significantly longer in patients with open thoracotomy [20]. The possible explanation for these results may be the individual differences in the patients’ lesions and the different proficiency of surgeons.

This study demonstrates that CLMs patients with a history of PI have a significant risk of higher rates of conversion to thoracotomy, which has been reported to have the disadvantages of scars, post-operative pain, long-term musculoskeletal morbidity, and vertebral scoliosis [21]. Differences in operative time, blood loss, chest tube placement time, length of stay, and postsurgical length of stay existed between the PI and NPI groups. Our findings are supported by those of previous retrospective studies [8, 12]. Elhattab and colleagues found a lower conversion rate to thoracotomy and postsurgical complications among asymptomatic patients than among patients with previous pulmonary infection [13]. Parish and colleagues also reported that postoperative complications were higher in infected lesions [5]. Other investigators have reported reduced conversion rates in patients without a history of pulmonary infection [4]. PI is more likely to present fibrovascular adhesions and give rise to difficulty in visualization, which may explain the discrepancies in the conversion rate between patients with and without PI in our study cohort. In addition, excision is technically challenging due to inflammatory changes such as lymph node enlargement, poor tissue plane definition, and neovascularization. Nonetheless, a few studies have not found differences. Aziz found that the complication rate was not statistically significant, although the length of stay was significantly higher in the symptomatic group [8].

However, these studies were limited by small sample sizes and may be confounded by covariates. It should be emphasized that the surgical outcome of patients with CLMs is affected by other factors in addition to previous pulmonary infection. As a result, these studies produced marked differences in the surgical outcomes of patients with PI or not. There were no randomized clinical trials or clinical practice guidelines to demonstrate the best management strategy in patients with asymptomatic CLMs. Our study has overcome many limitations of previous studies by propensity score matching and relatively large samples.

Our data show that patients with a history of pulmonary infection did make a difference in the outcome of the thoracoscopic procedure. In contrast, patients without a history of PI can be safely treated in thoracoscopic procedures without major postoperative complications. This study indicates that hesitation with operation intervention may lead to pulmonary infection. Such deferral of intervention may result in a missed opportunity to obtain an uneventful postoperative course. Moreover, subgroup analyses demonstrated that whether the time from successful medication to operation was more than one year was not associated with the conversion rate to thoracotomy, which may indicate the necessity of prophylactic surgical excision before symptoms appear in asymptomatic CLMs patients.

This study has several limitations. First, it is difficult to prove the relationship between those infections and the lesion. The definition of previous pulmonary infection in this study is mainly based on clinical symptoms and image analysis, which may not be correct precisely. Children with a lesion-induced pulmonary infection may not undergo chest radiography or CT. Moreover, it is difficult to ascertain infection episodes in some patients with recurrent infections, and patients in the non-infected group were relatively older after matching, which may cause selection bias. Second, although there was a relatively large sample of CLMs in this study, the number of patients in the matched cohort is still scant, and the conversion rate is too low to determine the impact factors. Third, this study only included patients who underwent elective operation and analysed the impact of previous pulmonary infection on the thoracoscopic procedure. However, patients who developed uncontrolled lung infections due to CLMs and who had to undergo emergency surgery as a result were not analysed in this study. Fourth, this was a single-center retrospective study, and future long-term follow-up studies are required to evaluate the effect of previous pulmonary infection on the outcome of thoracoscopic procedures in children with CLMs.

## Conclusions

In this observational study of patients with CLMs, a history of pulmonary infection was associated with an elevated risk of conversion to thoracotomy and postsurgical complications compared with non-infected patients, and thus they must be given careful perioperative management. Elective thoracoscopic procedures in asymptomatic CLMs patients are safe and effective, and operative intervention may be required before the onset of symptoms.

## Data Availability

The datasets used and/or analysed during the current study are available from the corresponding author on reasonable request.

## Abbreviations

CLMs: congenital lung malformations
PI: pulmonary infection
NPI: non-infection of pulmonary
PSM: propensity score matching
CT: computed tomography
